# Surgical management of a giant sternal chondromyxoid fibroma: a case report

**DOI:** 10.1186/s13019-015-0370-2

**Published:** 2015-11-28

**Authors:** Chen Chen, Xiaojie Huang, Mingjiu Chen, Fenglei Yu, Bangliang Yin, Yunchang Yuan

**Affiliations:** 1 Department of Thoracic Surgery, The Second Xiangya Hospital, Central South University, #139 Renmin Middle Rd, Changsha, Hunan 410011 P.R. China; 2 Department of Cardiovascular Surgery, The Second Xiangya Hospital, Central South University, Changsha, Hunan 410011 P.R. China

**Keywords:** Sternum, Reconstruction, Chondromyxoid fibroma, Titanium plate

## Abstract

**Background:**

A primary chondromyxoid fibroma (CMF) arising from sternum is quite uncommon tumor in thoracic surgery. Removal of giant sternal tumors requires extensive resection of the anterior chest wall, and results in deformity and paradoxical movement.

**Case Presentation:**

A 40-year-old female presented a progressively enlarging mass of her anterior chest wall. Computed tomography revealed an osteolytic lesion with discrete calcification in the bone marrow of the sternum. The tumor extended across the destroyed cortex to the parietal and visceral soft aspects, involving some of the costal cartilage and most of the sternal body. Partial sternal resection was performed successfully and an individual-specific stainless steel plate was used to reconstruct the anterior chest wall. The early result was good, however, nine months after the first surgery, fractures of plate were found at bilateral plate-clavicular junction. The plate had to be removed, and a titanium mesh was used to reconstruction of the chest wall. The patient has been of disease free for more than 18 month after the second surgery.

**Conclusions:**

Our experience indicated that the individual-specific plate may not be suitable for reconstructing both the anterior chest wall as well as the sternoclavicular joint after subtotal sternum resection.

## Background

A primary chondromyxoid fibroma (CMF) is an uncommon benign bone tumor which mainly affects the long bones and rarely occurs in the sternum [1–6]. Surgical removal of the sternum remains the first choice of treatment. Removal of giant sternal tumors requires extensive resection of the anterior chest wall, and results in large defects in bones and soft tissues, causing deformity and paradoxical movement. Although various prostheses, such as musculocutaneous flaps and alloplastic materials, have been used for chest wall reconstruction, the ultimate decision of how to reconstruct strongly depends on the surgeon’s experience [7–10]. We treated a patient who had a giant CMF arising from the sternum, with extensive resection which required two chest wall reconstruction surgeries.

## Case presentation

**Fig 1:**
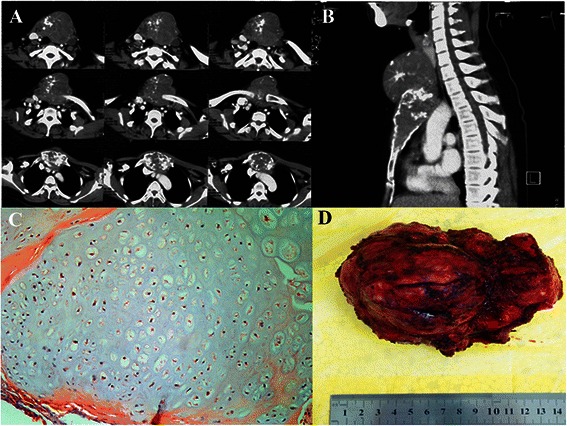
**a, b** The computed tomographic scan showed an expansive mass based on the sternal manubrium. The tumor extended across the destroyed cortex to the parietal and visceral soft aspects, involving some of the costal cartilage and most of the sternal body. **c, d** The size of the tumor was about 10 × 8 × 6 cm. The histological examination of the surgical specimen confirmed the diagnosis of chondromyxoid fibroma

A 40-year-old female was admitted to our hospital due to a progressively enlarging mass over four years of her anterior chest wall. Since October 2008, she had experienced intermittent anterior chest pain with the pain intensifying in August 2012. Physical examination revealed a warm 10 × 8 × 6 cm mass fixed to the upper sternum, and tender to palpation. No pulsation was noted. Computed tomography revealed an osteolytic lesion with discrete calcification in the bone marrow of the sternum. The tumor extended across the destroyed cortex to the parietal and visceral soft aspects, involving some of the costal cartilage and most of the sternal body (Fig. 1a, b).

**Fig 2:**
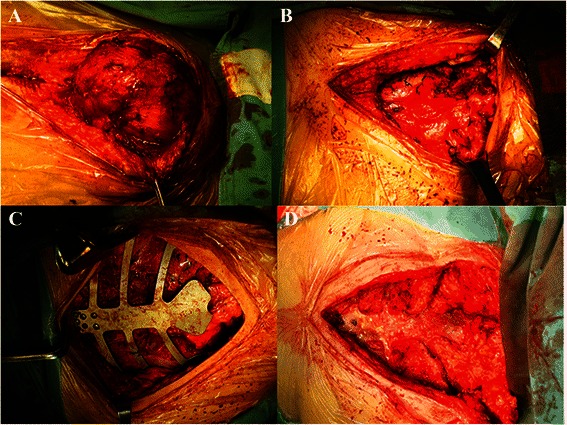
**a, b** The complete resection of the tumor left a defect measuring 18 × 15 cm^2^ on the anterior chest wall. **c** An individual-specific stainless steel plate was used to reconstruct the upper sternum, the costal arch and both sternoclavicular joints. **d** After the individual-specific plate was removed, pleural thickening was found at the anterior chest wall defect

**Fig 3:**
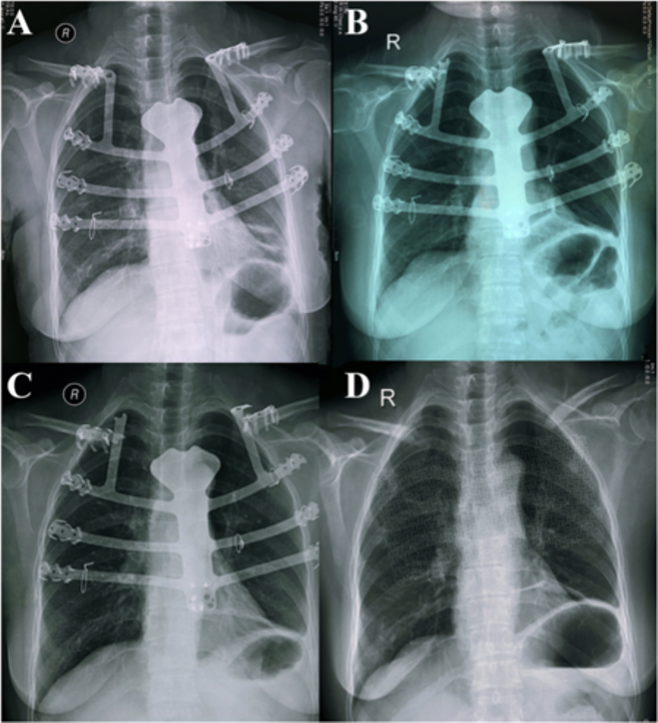
**a** X-ray showed the immediate postoperative result was favorable, since the individual-specific stainless steel plate was patient-specific, strong enough to protect the introthoracic cavity, and easy to adapt to the shape of the chest. **b** X-ray revealed displacement of the plate along the left 1st-3rd ribs and a fracture in the right plate-clavicular junction. **c** A similar plate-clavicular junction fracture developed on the left side. **d** Reconstruction of the chest wall was undertaken with a titanium mesh

During the surgery, the manubrium sterni, two-third of corpus sterni, both of the proximal clavicular heads as well as bilaterally the first three ribs and the costal arch with more than a 2.0 cm surgical margin were removed. Frozen section analysis of margins confirmed the complete resection. This resection left a defect measuring 18 × 15 cm^2^ on the anterior chest wall (Fig. 2a, b). According to the reconstructed images of the chest CT, an individual-specific stainless steel plate was made in the same shape as the thoracic bony structure of the patient, using for the reconstruction of the upper sternum, the costal arch and both sternoclavicular joints. The placement and fixation of the plate were straightforward without any difficulty. The securement of the plate was achieved with claw fixator and screws to the remaining ribs and clavicles (Fig. 2c, 3a). The surgery was successful, and the reconstruction of the chest wall was satisfactory both in appearance and function (Fig. 3a). The postoperative course was uneventful, and with a body belt, the patient was discharged on the 14th postoperative day. The histological examination of the surgical specimen confirmed the diagnosis of chondromyxoid fibroma (Fig. 1c, d).

However, nine months after the first surgery, the patient presented with aggravated chest pain and local plate exposure over the upper sternum. X-ray revealed displacement of the plate along the left 1st-3rd ribs and a fracture in the right plate-clavicular junction (Fig. 3b), two weeks later, a similar fracture developed on the left side (Fig. 3c). The complete surgical removal of the plate had to be performed through original incision. Reconstruction of the chest wall was then undertaken with a titanium mesh. The mesh was fixed to the manubrium and costal cartilage directly and pulled towards each rib stump. A soft tissue covering was sutured directly. Postoperatively, there was no paradoxical movement of the rib cage noted during respiration (Fig. 3d).

## Discussion

CMF of the sternum is one of the least commonly occurring bone tumors with only a few cases in the thoracic surgery literature describing the surgical treatment of this disease [1–6]. Although it is a benign tumor without malignant features histopathologically, pathological bone fractures and dysfunction can occur as the tumor grows larger. To avoid local recurrence, complete surgical resection, which usually requires wide excision and full-thickness resection, is strongly recommended. However, for a giant sternal tumor, complete resection may result in a large defect of the anterior chest wall requiring extensive reconstruction surgery to protect the intrathoracic organs [7–9, 11–14].

Various procedures have been described to reconstruct large defects of the anterior chest wall, such as musculocutaneous flaps [15], bone allografts [16], Gore-Tex dual mesh [17, 18], sandwiched polypropylene mesh [8] and titanium plating [19]. The ideal prosthetic material should be readily available, easy to use, durable, adaptable, resistant to infection and of low cost. However, most of the materials with these characteristics are clinically inadequate due to insufficient rigidity to protect introthoracic organs, being hard to handle or being difficult to conform to required shape of the patient’s chest. For our patient, the upper sternum, both clavicle proximal heads and bilaterally the first three ribs were removed in order to achieve a complete resection. Reconstructive surgery was needed to rebuild both the anterior chest wall and sternoclavicular joints. An individual-specific stainless steel plate was made and used for the first time to reconstruct the chest wall. The immediate postoperative result was favorable, since the individual-specific stainless steel plate was patient-specific, strong enough to protect the introthoracic cavity, and easy to adapt to the shape of the chest. However, the movement of the plate-clavicular junction over time, in the anteroposterior and vertical planes, rotationationally, as well as during actions of the shoulder girdle or scapula, caused a corresponding plate-clavicular junction fracture and displacement of the plate as early as nine months after the first surgery. This indicated that the individual-specific stainless steel plate might not be an adequate option for reconstruction of the anterior chest wall, especially given the need for stabilization of the plate-clavicular junction.

After the individual-specific plate was removed, the kind of prosthetic material to be used to reconstruct the chest wall needed to be considered. Girotti et al. developed a “rib-like” technique, a semi-rigid 3D prosthesis which could conform to the shape of the native ribs, and at the same time, protect the mediastinum, as well as allow a certain degree of freedom of movement for respiration [20]. Marulli and Hamad et al. described sternal replacement with allogenic cryopreserved sternum and costal cartilages, which was effective for geometrically covering the entire large anterior chest wall defect by using the same amount of bone and cartilages in the recipient [21]. Koto et al. reported the reconstruction with titanium mesh and transverse rectus abdominis myocutaneous flaps after subtotal sternal excision. This procedure was potentially applicable to extensive anterior chest wall resection, and had advantages compared with conventional prostheses such as its rigidity, flexibility, and usability [13]. During our surgery, after the individual-specific plate was removed, pleural thickening was found at the anterior chest wall defect. Since the pleura was thick, strong and flexible, it was considered to be a natural and perfect shield for the intrathoracic cavity (Fig. 2d). Thus, only a titanium mesh was used to supplement the closure of the anterior chest wall. The postoperative course was without complications.

## Conclusion

Our experience in this patient suggests that a CMF arising in the sternum is rare. A giant sternal CMF needs to be completely resected. An individual-specific plate may not be suitable for reconstructing both the anterior chest wall as well as the sternoclavicular joint after subtotal sternum resection. This is mainly due to the effect of respiratory movement of the anteroposterior chest wall on the plate and from vertical and rotation movements of the plate-clavicular junction. Titanium mesh remains our first choice to reconstruct the chest wall in these cases.

## Consent

Written informed consent was obtained from the patient for publication of this Case report and any accompanying images. A copy of the written consent is available for review by the Editor-in-Chief of this journal.
